# The asymptotic normality of internal estimator for nonparametric regression

**DOI:** 10.1186/s13660-018-1832-6

**Published:** 2018-09-10

**Authors:** Penghua Li, Xiaoqin Li, Liping Chen

**Affiliations:** 1 Automotive Electronics Engineering Research Center, College of Automation, Chongqing University of Posts and Telecommunications, Chongqing, China; 2 School of Mathematical Sciences, Anhui University, Hefei, China; 3 School of Electrical Engineering and Automation, Hefei University of Technology, Hefei, China

**Keywords:** 62G08, 62G20, Asymptotic normality, Nonparametric regression, Internal estimator, Dependent data

## Abstract

In this paper, we aim to study the asymptotic properties of internal estimator of nonparametric regression with independent and dependent data. Under some weak conditions, we present some results on asymptotic normality of the estimator. Our results extend some corresponding ones.

## Introduction

In this paper, we consider the nonparametric regression model

$$ Y_{i}=m(X_{i})+U_{i}, \quad 1\leq i\leq n,n \geq1, $$

where $(X_{i},Y_{i})\in R^{d}\times R$, $d\geq1$, and $U_{i}$ are random variables satisfying $E(U_{i}|X_{i})=0$, $1\leq i\leq n$, $n\geq1$. So we have

$$ E(Y_{i}|X_{i}=x)=m(x),\quad i\geq1. $$

Let $K(x)$ be a kernel function. Define $K_{h}(x)=h^{-d}K(x/h)$, where $h=h_{n}$ is a sequence of positive bandwidths tending to zero as $n\rightarrow\infty$. Kernel-type estimators of the regression function are widely used in various situations because of their flexibility and efficiency in the dependent and independent data. For the independent data, Nadaraya [1] and Watson [2] gave the most popular nonparametric estimator of the unknown function $m(x)$ named the Nadaraya–Watson estimator $\widehat{m}_{\mathrm{NW}}(x)$:

1.1 $$ \widehat{m}_{\mathrm{NW}}(x)=\frac{\sum_{i=1}^{n} Y_{i}K_{h}(x-X_{i})}{\sum_{i=1}^{n} K_{h}(x-X_{i})}. $$

Jones et al. [3] considered various versions of kernel-type regression estimators such as the Nadaraya–Watson estimator (1.1) and the local linear estimator. They also investigated the internal estimator

1.2 $$ \widehat{m}_{n}(x)=\frac{1}{n}\sum_{i=1}^{n} \frac{Y_{i}K_{h}(x-X_{i})}{f(X_{i})} $$

for a known density $f(\cdot)$. Here the factor $\frac{1}{f(X_{i})}$ is internal to the summation, whereas the estimator $\widehat{m}_{\mathrm{NW}}(x)$ has the factor $\frac{1}{\widehat{f}(x)}=\frac{1}{n^{-1}\sum_{i=1}^{n}K_{h}(x-X_{i})}$ externally to the summation.

The internal estimator was first proposed by Mack and Müller [4]. Jones et al. [3] studied various kernel-type regression estimators, including the introduced internal estimator (1.2). Linton and Nielsen [5] introduced an integration method based on direct integration of initial pilot estimator (1.2). Linton and Jacho-Chávez [6] studied the other internal estimator

1.3 $$ \widetilde{m}_{n}(x)=\frac{1}{n}\sum _{i=1}^{n} \frac{Y_{i}K_{h}(x-X_{i})}{\widehat{f}(X_{i})}, $$

where $\widehat{f}(X_{i})=\frac{1}{n}\sum_{j=1}^{n} L_{b}(X_{i}-X_{j})$ and $L_{b}(\cdot)=L(\cdot/b)/b^{d}$. Here $L(\cdot)$ is a kernel function, *b* is the bandwidth, and the density $f(\cdot)$ is unknown. Under the independent data, Linton and Jacho-Chávez [6] obtained the asymptotic normality of the internal estimator $\widetilde{m}_{n}(x)$ in (1.3). Shen and Xie [7] obtained the complete convergence and uniform complete convergence of internal estimator $\widehat{m}_{n}(x)$ in (1.2) under the geometrical *α*-mixing (or strong mixing) data. Li et al. [8] weakened the conditions of Shen and Xie [7] and obtained the convergence rate and uniform convergence rate for the estimator $\widehat{m}_{n}(x)$ in probability.

As far as we know, there are no results on asymptotic normality of the internal estimator $\widehat{m}_{n}(x)$. Similarly to Linton and Jacho-Chávez [6], we investigate the asymptotic normality of the internal estimator $\widehat{m}_{n}(x)$ with independent data and *φ*-mixing data, respectively. Asymptotic normality results are presented in Sect. 3.

Denote $\mathcal{F}_{n}^{m}=\sigma(X_{i}, n\leq i\leq m)$ and define the coefficients

$$ \varphi(n)=\sup_{m\geq1}\sup_{A\in\mathcal {F}_{1}^{m},B\in\mathcal {F}_{m+n}^{\infty},P(A)\neq0} \bigl\vert P(B|A)-P(B) \bigr\vert . $$

If $\varphi(n)\downarrow0$ as $n\rightarrow\infty$, then $\{X_{n}\}_{n\geq1}$ is said to be a *φ*-mixing sequence.

The concept of *φ*-mixing is introduced by Dobrushin [9], and many properties of *φ*-mixing are presented in Chap. 4 of Billingsley [10]. If the coefficient of the process is geometrically decreasing, then the autoregressive moving average (ARMA) process can construct a geometric *φ*-mixing sequence. Györfi et al. [11, 12] gave more examples and applications to nonparametric estimation. We can also refer to Fan and Yao [13] and Bosq and Blanke [14] for the works on nonparametric regression under independent and dependent data.

Regarding notation, for $x=(x_{1},\ldots,x_{d})\in R^{d}$, set $\|x\|=\max(|x_{1}|,\ldots,|x_{d}|)$. Throughout the paper, $c,c_{1},c_{2},c_{3},\ldots,d,B_{0},B_{1}$ denote some positive constants not depending on *n*, which may be different in various places, $\lfloor x\rfloor$ denotes the largest integer not exceeding *x*, → means to take the limit as $n\rightarrow\infty$, and $c_{n}\sim d_{n}$ means that $\frac{c_{n}}{d_{n}}\rightarrow1$, $\xrightarrow {\mathscr{D}}$ means the convergence in distribution, and $X\stackrel{\mathscr{D}}{=}Y$ means that random variables *X* and *Y* have the same distribution. A sequence $\{X_{i},i\geq1\}$ is said to be second-order stationary if $(X_{1},X_{1+k})\stackrel{\mathscr{D}}{=} (X_{i},X_{i+k})$ for $i\geq1$, $k\geq1$.

## Some assumptions

In this section, we list some assumptions.

### Assumption 2.1

There exist two positive constants $\bar{K}>0$ and $\mu>0$ such that

2.1 $$ \sup_{u\in R^{d}} \bigl\vert K(u) \bigr\vert \leq \bar{K} \quad \text{and}\quad \int_{R^{d}} \bigl\vert K(u) \bigr\vert \,du= \mu. $$

### Assumption 2.2

Let $S_{f}$ denote the compact support of known density $f(\cdot)$ of $X_{1}$. For $x\in S_{f}$, the function $m(x)$ is twice differentiable, and there exists a positive constant *b* such that

$$\biggl\vert \frac{\partial^{2} m(x)}{\partial x_{i}\, \partial x_{j}} \biggr\vert \leq b,\quad \forall i,j=1,2, \ldots,d. $$

The kernel density function is symmetric and satisfies

$$\int_{R^{d}}|v_{i}||v_{j}|K(v)\,dv< \infty, \quad \forall i,j=1,2,\ldots,d. $$

### Assumption 2.3

We assume the data observed $\{(X_{i},Y_{i}),i\geq1\}$ is an independent and identically distributed stochastic sequence with values in $R^{d}\times R$. The known density $f(\cdot)$ of $X_{1}$ is upon its compact support $S_{f}$ and such that $\inf_{x\in S_{f}}f(x)>0$. For $0<\delta\leq1$, we suppose that

2.2 $$ E|Y_{1}|^{2+\delta}< \infty $$

and

2.3 $$ \sup_{x\in S_{f}} E\bigl(|Y_{1}|^{2+\delta}|X_{1}=x \bigr)f(x)\leq B_{0}< \infty. $$

### Assumption 2.3^∗^

We assume that the data observed $\{(X_{i},Y_{i}),i\geq1\}$ is a second-order stationary stochastic sequence with values in $R^{d}\times R$. The sequence $\{(X_{i},Y_{i}),i\geq1\}$ is also assumed to be *φ*-mixing with $\sum_{n=1}^{\infty}\varphi^{1/2}(n)<\infty$. The known density $f(\cdot)$ of $X_{1}$ is upon its compact support $S_{f}$ and such that $\inf_{x\in S_{f}}f(x)>0$. Let (2.2) and (2.3) be fulfilled. Moreover, for all $j\geq1$, we have

2.4 $$\begin{aligned} \sup_{x_{1}\in S_{f},x_{j+1}\in S_{f}} E\bigl( \vert Y_{1}Y_{j+1} \vert |X_{1}=x_{1},X_{j+1}=x_{j+1} \bigr)f_{j}(x_{1},x_{j+1})\leq B_{1}< \infty, \end{aligned}$$

where $f_{j}(x_{1},x_{j+1})$ denotes the joint density of $(X_{1},X_{j+1})$.

### Remark 2.1

Assumption 2.1 is a usual condition on the kernel function, and Assumption 2.2 is used to get the convergence rate of $|E\widehat{m}_{n}(x)-m(x)|$. Assumptions 2.3 and 2.3^∗^ are the conditions of independent and dependent data $\{(X_{i},Y_{i}),i\geq 1\}$, respectively. Similarly to Hansen [15], conditions (2.2) and (2.3) are used to control the tail behavior of the conditional expectation $E(|Y_{1}|^{2+\delta}|X_{1}=x)$, and (2.4) is used to estimate the covariance $\operatorname{Cov}(Y_{1},Y_{j+1})$.

## Asymptotic normality of internal estimator $\widehat{m}_{n}(x)$ with independent and dependent data

In this section, we show some results on asymptotic normality of the internal estimator of a nonparametric regression model with independent and dependent data. Theorem 3.1 is for independent data, and Theorem 3.2 is for *φ*-mixing data.

### Theorem 3.1

*Let Assumptions* 2.1*–*2.3 *hold*, *and let* $\lim_{\|u\|\rightarrow\infty}\|u\|^{d}K(u)=0$. *Suppose that* $\frac{E(Y_{1}^{2}|X_{1}=x)}{f(x)}$ *is positive and continuous at point* $x\in S_{f}$. *If* $0< h^{d}\rightarrow0$, $nh^{d}\rightarrow\infty$, *and* $nh^{d+4}\rightarrow0$ *as* $n\rightarrow\infty$, *then*

3.1 $$ \sqrt{nh^{d}}\bigl[\widehat{m}_{n}(x)-m(x)\bigr] \xrightarrow{~~\mathscr{D}~~}N\bigl(0,\sigma ^{2}(x) \bigr), $$

*where* $\sigma^{2}(x)=\frac{E(Y_{1}^{2}|X_{1}=x)}{f(x)}\int_{R^{d}} K^{2}(u)\,du$.

### Theorem 3.2

*Let the conditions of Theorem *3.1 *be fulfilled*, *where Assumption *2.3 *is replaced by Assumption *2.3^∗^. *Then* (3.1) *holds*.

### Remark 3.1

The choice of a positive bandwidth *h* is easy to design. For example, with $d\geq1$, if $h=n^{-\beta}$ and $\beta\in (\frac{1}{d+4},\frac{1}{d})$, then the conditions $0< h^{d}\rightarrow0$, $nh^{d}\rightarrow\infty$, and $nh^{d+4}\rightarrow0$ are satisfied as $n\rightarrow\infty$.

## Conclusion

Linton and Jacho-Chávez [6] obtained some asymptotic normality results of the internal estimator $\widetilde{m}_{n}(x)$ under independent data. Comparing Theorem 1 and Corollary 1 of Linton and Jacho-Chávez [6], our asymptotic normality results on the internal estimator $\widehat{m}_{n}(x)$ in Theorems 3.1 and 3.2 are relatively simple. Meanwhile, we use the method of Bernstein’s big-block and small-block and the inequalities of *φ*-mixing random variables to investigate the asymptotic normality of the internal estimator $\widehat{m}_{n}(x)$ for $m(x)$, and we also obtain the asymptotic normality result of (3.1). Obviously, *α*-mixing is weaker than *φ*-mixing, but some moment inequalities of *α*-mixing are more complicated than those of *φ*-mixing [16, 17]. For simplicity, we study the asymptotic normality of internal estimator $\widehat{m}_{n}(x)$ under *φ*-mixing and obtain the asymptotic normality result of Theorem 3.2.

## Some lemmas and the proofs of main results

### Lemma 5.1

(Liptser and Shiryayev [18], Theorem 9 in Sect. 5)

*Let* $(\xi_{nk},\mathscr{H}_{k}^{n})_{k\geq1}$ *be martingale differences* (*i*.*e*. $\mathscr{H}_{0}^{n}=\{\emptyset,\Omega\}$, $\mathscr{H}_{k}^{n} \subset\mathscr{H}_{k+1}^{n}$, $\xi_{nk}$ *is an* $\mathscr{H}_{k}^{n}$-*measurable random variable*, $E(\xi_{nk}|\mathscr{H}_{k-1}^{n})=0$ *a*.*s*., *for all* $k\geq1$ *and* $n\geq1$) *with* $E\xi_{nk}^{2}<\infty$ *for all* $k\geq1$ *and* $n\geq1$. *Let* $(\gamma _{n})_{n\geq1}$ *be a sequence of Markov times with respect to* $(\mathscr {H}_{k}^{n})_{k\geq0}$, *taking values in the set* $\{0,1,2,\ldots\}$. *If*

$$\begin{aligned}& \sum_{k=1}^{\gamma_{n}}E\bigl(\xi_{nk}^{2}I \bigl( \vert \xi_{nk} \vert >\delta\bigr)|\mathscr {H}_{k-1}^{n}\bigr)\xrightarrow{~~P~~}0,\quad \forall\delta\in (0,1], \\& \sum_{k=1}^{\gamma_{n}}E\bigl(\xi_{nk}^{2}| \mathscr{H}_{k-1}^{n}\bigr)\xrightarrow{~~P~~} \sigma^{2}, \end{aligned}$$

*then*

$$ \sum_{k=1}^{\gamma_{n}}\xi_{nk} \xrightarrow{~~\mathscr{D}~~}N\bigl(0,\sigma ^{2}\bigr). $$

### Lemma 5.2

(Billingsley [10], Lemma 1)

*If* *ξ* *is measurable with respect to* $\mathscr{M}^{k}_{-\infty}$ *and* *η* *is measurable with respect to* $\mathscr{M}^{\infty}_{k+n}$ ($n\geq0$), *then*

$$E|\xi|^{r}< \infty,\qquad E|\eta|^{s}< \infty, \quad r,s>1, r^{-1}+s^{-1}=1, $$

*implies*

$$ \bigl\vert E(\xi\eta)-E(\xi)E(\eta) \bigr\vert \leq 2\varphi^{\frac{1}{r}}(n) \bigl(E \vert \xi \vert ^{r}\bigr)^{\frac{1}{r}}\bigl(E \vert \eta \vert ^{s}\bigr)^{\frac {1}{s}}. $$

### Lemma 5.3

(Yang [16], Lemma 2)

*Let* $p\geq2$, *and let* $\{X_{n}\}_{n\geq1}$ *be a* *φ*-*mixing sequence with* $\sum_{n=1}^{\infty}\varphi^{1/2}(n)<\infty$. *If* $EX_{n}=0$ *and* $E|X_{n}|^{p}<\infty$ *for all* $n\geq1$, *then*

$$ E \Biggl\vert \sum_{i=1}^{n} X_{i} \Biggr\vert ^{p}\leq C \Biggl(\sum _{i=1}^{n} E|X_{i}|^{p}+ \Biggl( \sum_{i=1}^{n} EX_{i}^{2} \Biggr)^{p/2} \Biggr), $$

*where* *C* *is a positive constant depending only on* $\varphi(\cdot)$.

### Lemma 5.4

(Fan and Yao [13], Proposition 2.6)

*Let* $\mathscr{F}_{i}^{j}$ *and* $\alpha(\cdot)$ *be the same as in* (2.57) *of Fan and Yao* [13]. *Let* $\xi_{1},\xi_{2},\ldots,\xi_{k}$ *be complex*-*valued random variables measurable with respect to the* *σ*-*algebras* $\mathscr{F}_{i_{1}}^{j_{1}},\ldots,\mathscr{F}_{i_{k}}^{j_{k}}$, *respectively*. *Suppose* $i_{l+1}-j_{l}\geq n$ *for* $l=1,\ldots,k-1$ *and* $j_{l}\geq i_{l}$ *and* $P(|\xi_{l}|\leq1)=1$ *for* $l=1,2,\ldots,k$. *Then*

$$ \bigl\vert E(\xi_{1}\cdots\xi_{k})-E(\xi_{1}) \cdots E(\xi_{k}) \bigr\vert \leq 16(k-1)\alpha(n). $$

### Proof of Theorem 3.1

It is easy to see that

5.1 $$ \sqrt{nh^{d}} \bigl(\widehat{m}_{n}(x)-m(x) \bigr)= \sqrt{nh^{d}} \bigl(\bigl[\widehat {m}_{n}(x)-E \widehat{m}_{n}(x)\bigr]+\bigl[E\widehat{m}_{n}(x)-m(x)\bigr] \bigr). $$

Combining Assumption 2.2 with the proof of Lemma 2 of Shen and Xie [7], we obtain that

$$\bigl\vert E\widehat{m}_{n}(x)-m(x) \bigr\vert =O \bigl(h^{2}\bigr),\quad x\in S_{f}. $$

Then, it follows from $nh^{d+4}\rightarrow0$ that

5.2 $$ \sqrt{nh^{d}}\bigl[E\widehat{m}_{n}(x)-m(x)\bigr]=O\bigl( \sqrt{nh^{d+4}}\bigr)\rightarrow 0, \quad x\in S_{f}. $$

For $x\in S_{f}$, let $Z_{i}:=\sqrt{h^{d}}\frac{Y_{i}K_{h}(x-X_{i})}{f(X_{i})}$, $1\leq i\leq n$. Denote

5.3 $$\begin{aligned} \sqrt{nh^{d}}\bigl[\widehat{m}_{n}(x)-E\widehat{m}_{n}(x) \bigr] =&\frac{1}{\sqrt {n}}\sum_{i=1}^{n} \sqrt{h^{d}} \biggl[\frac{Y_{i}K_{h}(x-X_{i})}{f(X_{i})}-E\frac{Y_{i}K_{h}(x-X_{i})}{f(X_{i})} \biggr] \\ =&\frac{1}{\sqrt{n}}\sum_{i=1}^{n} (Z_{i}-EZ_{i}). \end{aligned}$$

To prove (3.1), we apply (5.1)–(5.3) and have to show that

5.4 $$ \sqrt{nh^{d}}\bigl[\widehat{m}_{n}(x)-E\widehat{m}_{n}(x) \bigr]=\frac{1}{\sqrt{n}}\sum_{i=1}^{n} (Z_{i}-EZ_{i})\xrightarrow{~~\mathscr{D}~~}N\bigl(0, \sigma^{2}(x)\bigr), $$

where $\sigma^{2}(x)$ is defined by (3.1).

Combining the independent and identically distributed stochastic sequence of $\{(X_{i},Y_{i}), i\geq1\}$ with Lemma 5.1, to prove (5.4), we have to show that

5.5 $$ \frac{1}{n}\sum_{i=1}^{n} E(Z_{i}-EZ_{i})^{2}=\operatorname{Var} (Z_{1})\rightarrow\sigma^{2}(x) $$

and, for all $\lambda\in(0,1]$,

5.6 $$ \frac{1}{n}\sum_{i=1}^{n} E \biggl((Z_{i}-EZ_{i})^{2}I \biggl( \frac{|Z_{i}-EZ_{i}|}{\sqrt{n}}>\lambda \biggr) \biggr)\rightarrow0. $$

Obviously, for any $1\leq r\leq2+\delta$ ($0<\delta\leq1$), by (2.1) and (2.3) we have

5.7 $$\begin{aligned}& h^{d(r-1)}E \biggl\vert \frac{K_{h}(x-X_{1})Y_{1}}{f(X_{1})} \biggr\vert ^{r} \\& \quad = h^{d(r-1)}E \biggl(\frac{|K_{h}(x-X_{1})|^{r}}{f^{r}(X_{1})}E\bigl(|Y_{1}|^{r}|X_{1} \bigr) \biggr) \\& \quad = \int_{S_{f}} \biggl\vert K\biggl(\frac{x-u}{h}\biggr) \biggr\vert ^{r}E\bigl(|Y_{1}|^{r}|X_{1}=u \bigr)\frac{1}{h^{d}}\frac {f(u)}{f^{r}(u)}\,du \\& \quad \leq \int_{S_{f}} \biggl\vert K\biggl(\frac{x-u}{h}\biggr) \biggr\vert ^{r}\bigl(E\bigl(|Y_{1}|^{2+\delta }|X_{1}=u \bigr)f(u)\bigr)^{\frac{r}{2+\delta}}\frac{1}{h^{d}}\frac{1}{f^{\frac {(3+\delta)r}{2+\delta}-1}(u)}\,du \\& \quad \leq \frac{(B_{0})^{\frac{r}{2+\delta}}\bar{K}^{r-1}\mu}{(\inf_{x\in S_{f}} f(x))^{\frac{(3+\delta)r}{2+\delta}-1}}:=\bar{\mu}(r)< \infty. \end{aligned}$$

By (5.7) with $r=1$ this yields

5.8 $$ (EZ_{1})^{2}=h^{d} \biggl(E\frac{K_{h}(x-X_{1})Y_{1}}{f(X_{1})} \biggr)^{2} \leq ch^{d}\rightarrow0. $$

Define

$$ g(x)= \textstyle\begin{cases} \frac{E(Y_{1}^{2}|X_{1}=x)}{f(x)} &\text{if } x\in S_{f}, \\ 0 &\text{otherwise}. \end{cases} $$

In view of condition (2.3), we have

$$\begin{aligned} \int_{R^{d}}g(x)\,dx =& \int_{S_{f}}\frac{E(Y_{1}^{2}|X_{1}=x)}{f(x)}\,dx= \int _{S_{f}}\frac{E(Y_{1}^{2}|X_{1}=x)f^{\frac{2}{2+\delta}}(x)}{f^{\frac{4+\delta }{2+\delta}}(x)}\,dx \\ \leq& \int_{S_{f}}\frac{(E(|Y_{1}|^{2+\delta}|X_{1}=x))^{\frac{2}{2+\delta }}f^{\frac{2}{2+\delta}}(x)}{f^{\frac{4+\delta}{2+\delta }}(x)}\,dx \\ \leq& \frac{B_{0}^{\frac{2}{2+\delta}}}{(\inf_{x\in S_{f}} f(x))^{\frac{6+2\delta}{2+\delta}}} \int_{R^{d}} f(x)\,dx=\frac{B_{0}^{\frac{2}{2+\delta}}}{(\inf_{x\in S_{f}} f(x))^{\frac{6+2\delta}{2+\delta}}}< \infty. \end{aligned}$$

So we have $g(x)\in L_{1}$. Since that $\frac{E(Y_{1}^{2}|X_{1}=x)}{f(x)}$ is positive and continuous at a point $x\in S_{f}$ and $\lim_{\|u\|\rightarrow\infty}\|u\|^{d}K(u)=0$, we obtain by Bochner lemma [14] that

5.9 $$\begin{aligned} E\bigl(Z_{1}^{2}\bigr) =&h^{d}E \biggl( \frac{K_{h}(x-X_{1})Y_{1}}{f(X_{1})} \biggr)^{2}= \int _{S_{f}}K^{2}\biggl(\frac{x-u}{h}\biggr)E \bigl(Y_{1}^{2}|X_{1}=u\bigr)\frac{1}{h^{d}} \frac {1}{f(u)}\,du \\ =& \int_{R^{d}}K^{2}\biggl(\frac{x-u}{h}\biggr) \frac{1}{h^{d}}g(u)\,du \rightarrow \frac{E(Y_{1}^{2}|X_{1}=x)}{f(x)} \int_{R^{d}} K^{2}(u)\,du. \end{aligned}$$

Then, it follows from (5.8) and (5.9) that, for $x\in S_{f}$,

5.10 $$ \operatorname{Var}(Z_{1})\rightarrow \frac{E(Y_{1}^{2}|X_{1}=x)}{f(x)} \int_{R^{d}} K^{2}(u)\,du=\sigma^{2}(x), $$

which implies (5.6). Meanwhile, for some $\delta\in(0,1]$ and any $\lambda\in(0,1]$, by $C_{r}$ inequality and (5.7) we get that

5.11 $$\begin{aligned} \frac{1}{n}\sum_{i=1}^{n} E \biggl((Z_{i}-EZ_{i})^{2}I \biggl( \frac{|Z_{i}-EZ_{i}|}{\sqrt{n}}>\lambda \biggr) \biggr) =&E \biggl((Z_{1}-EZ_{1})^{2}I \biggl(\frac{|Z_{1}-EZ_{1}|}{\sqrt{n}}>\lambda \biggr) \biggr) \\ \leq&\frac{1}{n^{\frac{\delta}{2}}\lambda^{\delta }}E|Z_{1}-EZ_{1}|^{2+\delta} \leq \frac{c_{1}}{n^{\frac{\delta}{2}}\lambda^{\delta}}E|Z_{1}|^{2+\delta } \\ \leq&\frac{c_{2}}{(nh^{d})^{\frac{\delta}{2}}}\rightarrow0, \end{aligned}$$

since $nh^{d}\rightarrow\infty$. Thus, (5.6) follows from (5.11). Consequently, the proof of the theorem is completed. □

### Proof of Theorem 3.2

We use the same notation as in the proof of Theorem 3.1. Under the conditions of Theorem 3.2, by (5.1), (5.2), and (5.3), to prove (3.1), we need to show that

5.12 $$ \sqrt{nh^{d}}\bigl[\widehat{m}_{n}(x)-E\widehat{m}_{n}(x) \bigr]=\frac{1}{\sqrt{n}}\sum_{i=1}^{n} (Z_{i}-EZ_{i})\xrightarrow{~~\mathscr{D}~~}N\bigl(0, \sigma^{2}(x)\bigr), $$

where $\sigma^{2}(x)$ is defined by (3.1). By the second-order stationarity, $\{(X_{i},Y_{i}),i\geq1\}$ are identically distributed. Then, for $1\leq i\leq n$, we have by (5.8) and (5.9) that

5.13 $$ \operatorname{Var}(Z_{i}-EZ_{i})=\operatorname{Var}(Z_{1}) \rightarrow\frac {E(Y_{1}^{2}|X_{1}=x)}{f(x)} \int_{R^{d}} K^{2}(u)\,du=\sigma^{2}(x). $$

For $j\geq1$, in view of (2.4), we have

5.14 $$\begin{aligned}& E \biggl\vert \frac{K_{h}(x-X_{1})K_{h}(x-X_{j+1})Y_{1}Y_{j+1}}{f(X_{1})f(X_{j+1})} \biggr\vert \\& \quad = E \biggl(\frac{|K_{h}(x-X_{1})K_{h}(x-X_{j+1})|}{f(X_{1})f(X_{j+1})} E\bigl( \vert Y_{1}Y_{j+1} \vert |X_{1},X_{j+1}\bigr) \biggr) \\& \quad = \int_{S_{f}} \int_{S_{f}} \biggl\vert K\biggl(\frac{x-u_{1}}{h}\biggr)K \biggl(\frac{x-u_{j+1}}{h}\biggr) \biggr\vert E\bigl( \vert Y_{1}Y_{j+1} \vert |X_{1}=u_{1},X_{j}=u_{j+1} \bigr) \\& \qquad {} \times\frac{1}{h^{2d}}\frac {1}{f(u_{1})f(u_{j+1})}f_{j}(u_{1},u_{j+1}) \,du_{1}\,du_{j+1} \\& \quad \leq \frac{B_{1}}{(\inf_{x\in S_{f}} f(x))^{2}} \int_{R^{d}} \int_{R^{d}} \biggl\vert K\biggl(\frac{x-u_{1}}{h}\biggr)K \biggl(\frac {x-u_{j+1}}{h}\biggr) \biggr\vert \frac{1}{h^{2d}} \,du_{1}\,du_{j+1} \\& \quad \leq \frac{B_{1}\mu^{2}}{(\inf_{x\in S_{f}} f(x))^{2}}< \infty. \end{aligned}$$

So it follows from (5.7) and (5.14) that

5.15 $$ \bigl\vert \operatorname{Cov}(Z_{1},Z_{j}) \bigr\vert \leq E \vert Z_{1}Z_{j} \vert +\bigl(E \vert Z_{1} \vert \bigr)^{2}\leq c_{1}h^{d},\quad j>1. $$

Obviously, by the stationarity we establish that

5.16 $$\begin{aligned} \frac{1}{n}\operatorname{Var} \Biggl(\sum_{i=1}^{n} (Z_{i}-EZ_{i}) \Biggr) =&\frac{1}{n} \operatorname{Var} \Biggl(\sum_{i=1}^{n} Z_{i} \Biggr)=\operatorname{Var}(Z_{1})+\frac{2}{n} \sum_{1\leq i< j\leq n}\operatorname{Cov} (Z_{i},Z_{j}) \\ =&\operatorname{Var}(Z_{1})+\frac{2}{n} \biggl\{ \biggl[\sum _{\substack{1\leq i< j\leq n\\1\leq j-i\leq r_{n}}} +\sum_{\substack{1\leq i< j\leq n\\j-i>r_{n}}} \biggr]\operatorname{Cov} (Z_{i},Z_{j}) \biggr\} . \end{aligned}$$

For $h^{d}$, we can choose $r_{n}$ satisfying that $r_{n}\rightarrow\infty$ and $h^{d}r_{n}\rightarrow0$ as $n\rightarrow\infty$. So, by (5.15),

5.17 $$ \frac{2}{n}\sum_{\substack{1\leq i< j\leq n\\1\leq j-i\leq r_{n}}} \bigl\vert \operatorname{Cov} (Z_{i},Z_{j}) \bigr\vert \leq ch^{d}r_{n}\rightarrow0. $$

By Lemma 5.2 with $s=r=2$, the condition $\sum_{n=1}^{\infty}\varphi^{1/2}(n)<\infty$, and (5.9), we can show that

5.18 $$ \frac{2}{n}\sum_{\substack{1\leq i< j\leq n\\j-i>r_{n}}} \bigl\vert \operatorname{Cov} (Z_{i},Z_{j}) \bigr\vert \leq \frac{c_{1}}{n}\sum_{\substack{1\leq i< j\leq n\\j-i>r_{n}}}\varphi^{1/2}(j-i) \leq c_{2}\sum_{k>r_{n}}\varphi^{1/2}(k) \rightarrow0. $$

Therefore, by (5.13), (5.16), (5.17), and (5.18), we get that

$$ \frac{1}{n}\operatorname{Var} \Biggl(\sum_{i=1}^{n} Z_{i} \Biggr)= \sigma^{2}(x) \bigl(1+o(1)\bigr). $$

Next, we employ Bernstein’s big-block and small-block procedure (see Fan and Yao [13] and Masry [19]). Partition the set $\{1,2,\ldots,n\}$ into $2k_{n}+1$ subsets with large block of size $\mu=\mu_{n}$ and small block of size $\nu=\nu_{n}$ and set

5.19 $$ k=k_{n}=\biggl\lfloor \frac{n}{\mu_{n}+\nu_{n}}\biggr\rfloor . $$

Define $\mu=\mu_{n}=\lfloor\sqrt{\frac{n}{h^{d}}}\rfloor$ and $\nu=\nu_{n}=\lfloor\sqrt{nh^{d}}\rfloor$. So we have by $h^{d}\rightarrow0$ and $nh^{d}\rightarrow\infty$ that

5.20 $$ \begin{aligned} &\mu_{n}\rightarrow\infty,\qquad \nu_{n}\rightarrow\infty, \qquad \frac{\mu _{n}}{n}\rightarrow0, \\ &\frac{\nu_{n}}{n}\rightarrow0,\qquad \frac{\nu_{n}}{\mu_{n}}\rightarrow0,\qquad k_{n}=O\bigl( \sqrt{nh^{d}}\bigr). \end{aligned} $$

Define $\eta_{j}$, $\xi_{j}$, and $\zeta_{j}$ as follows:

5.21 $$\begin{aligned}& \eta_{j} := \sum_{i=j(\mu+\nu)+1}^{j(\mu+\nu)+\mu}(Z_{i}-EZ_{i}), \quad 0\leq j\leq k-1, \end{aligned}$$

5.22 $$\begin{aligned}& \xi_{j} := \sum_{i=j(\mu+\nu)+\mu+1}^{(j+1)(\mu+\nu)}(Z_{i}-EZ_{i}), \quad 0\leq j\leq k-1, \end{aligned}$$

5.23 $$\begin{aligned}& \zeta_{k} := \sum_{i=k(\mu+\nu)+1}^{n}(Z_{i}-EZ_{i}) . \end{aligned}$$

In view of

5.24 $$ S_{n}:=\sum_{i=1}^{n} (Z_{i}-EZ_{i})=\sum_{j=0}^{k-1} \eta_{j}+\sum_{j=0}^{k-1} \xi_{j}+\zeta _{k}:=S_{n}^{\prime}+S_{n}^{\prime\prime} +S_{n}^{\prime\prime\prime}, $$

we have to show that

5.25 $$\begin{aligned}& \frac{1}{n}E\bigl(S_{n}^{\prime\prime}\bigr)^{2} \rightarrow0,\qquad \frac {1}{n}E\bigl(S_{n}^{\prime\prime\prime} \bigr)^{2}\rightarrow0, \end{aligned}$$

5.26 $$\begin{aligned}& \Biggl\vert E \bigl(\exp\bigl(itn^{-1/2}S_{n}^{\prime} \bigr) \bigr)-\prod_{j=0}^{k-1}E \bigl(\exp \bigl(itn^{-1/2}\eta_{j}\bigr) \bigr) \Biggr\vert \rightarrow0, \end{aligned}$$

5.27 $$\begin{aligned}& \frac{1}{n}\sum_{j=0}^{k-1}E\bigl( \eta_{j}^{2}\bigr)\rightarrow\sigma^{2}(x), \end{aligned}$$

5.28 $$\begin{aligned}& \frac{1}{n}\sum_{j=0}^{k-1}E\bigl( \eta_{j}^{2}I\bigl( \vert \eta_{j} \vert > \varepsilon\sigma (x)\sqrt{n}\bigr)\bigr)\rightarrow0, \quad \forall \varepsilon>0. \end{aligned}$$

Relation (5.25) implies that $\frac{S_{n}^{\prime\prime}}{\sqrt{n}}$ and $\frac{S_{n}^{\prime\prime\prime}}{\sqrt{n}}$ are asymptotically negligible, (5.26) shows that the summands $\{\eta_{j}\}$ in $S_{n}^{\prime}$ are asymptotically independent, and (5.27)–(5.28) are the standard Lindeberg–Feller conditions for the asymptotic normality of $S_{n}^{\prime}$ under independence.

First, we prove (5.25). By (5.22) and (5.24) we have

5.29 $$ E\bigl(S_{n}^{\prime\prime}\bigr)^{2}=\operatorname{Var} \Biggl(\sum_{j=0}^{k-1}\xi_{j} \Biggr)=\sum_{j=0}^{k-1}\operatorname{Var}( \xi_{j})+ 2\sum_{0\leq i< j\leq k-1}\operatorname{Cov}( \xi_{i},\xi_{j}):=F_{1}+F_{2}. $$

By the stationarity and (5.10), similarly to the proof of (5.17) and (5.18), for $0\leq j\leq k-1$, we have

5.30 $$ \operatorname{Var}(\xi_{j})=\nu_{n}\operatorname{Var}(Z_{1})+2 \sum_{1\leq i< j\leq\nu_{n}}\operatorname{Cov}(Z_{i},Z_{j})= \nu_{n}\sigma^{2}(x) \bigl(1+o(1)\bigr). $$

Thus it follows from (5.19) and (5.20) that

5.31 $$ F_{1}=k_{n}\nu_{n}\sigma^{2}(x) \bigl(1+o(1)\bigr)\sim\frac{n\nu_{n}}{\mu_{n}+\nu_{n}}\sim\frac {n\nu_{n}}{\mu_{n}}=o(n). $$

We consider the term $F_{2}$ in (5.29). With $\lambda_{j}=j(\mu_{n}+\nu_{n})+\mu_{n}$,

$$ F_{2}=2\sum_{0\leq i< j\leq k-1}\sum _{l_{1}=1}^{\nu_{n}}\sum_{l_{2}=1}^{\nu_{n}} \operatorname{Cov}(Z_{\lambda_{i}+l_{1}},Z_{\lambda_{j}+l_{2}}), $$

but since $i\neq j$, $|\lambda_{i}-\lambda_{j}+l_{1}-l_{2}|\geq\mu_{n}$ for $0\leq i< j\leq k-1$, $1\leq l_{1}\leq\nu_{n}$, and $1\leq l_{2}\leq\nu_{n}$, similarly to the proof of (5.18), it follows that

5.32 $$ |F_{2}|\leq2\sum_{\substack{1\leq i< j\leq n \\ j-i\geq\mu_{n}}} \bigl\vert \operatorname{Cov}(Z_{i},Z_{j}) \bigr\vert =o(n). $$

Hence by (5.29), (5.31), and (5.32) we have

$$\frac{1}{n}E\bigl(S_{n}^{\prime\prime}\bigr)^{2} \rightarrow0. $$

By (5.13), (5.20), and (5.23), similarly to the proofs of (5.17) and (5.18), we can find that

$$\begin{aligned} \frac{1}{n}E\bigl(S_{n}^{\prime\prime\prime}\bigr)^{2} \leq& \frac{1}{n}\bigl(n-k_{n}(\mu_{n}+\nu_{n}) \bigr)\operatorname{Var} (Z_{1})+\frac{2}{n}\sum _{1\leq i< j\leq n-k_{n}(\mu_{n}+\nu_{n})} \bigl\vert \operatorname{Cov}(Z_{i},Z_{j}) \bigr\vert \\ \leq&C\frac{\mu_{n}+\nu_{n}}{n}\sigma^{2}(x)+o(1)\rightarrow0. \end{aligned}$$

Thus

5.33 $$ \frac{1}{\sqrt{n}}S_{n}=\frac{1}{\sqrt{n}}\bigl(S_{n}^{\prime}+S_{n}^{\prime\prime }+S_{n}^{\prime\prime\prime} \bigr) =\frac{1}{\sqrt{n}}S_{n}^{\prime}+o_{p}(1). $$

Second, it is easy to see that $\varphi^{1/2}(n)=o(\frac{1}{n})$ by $\varphi(n)\downarrow0$ and $\sum_{n=1}^{\infty}\varphi^{1/2}(n)<\infty$. Note that $\eta_{a}$ is $\mathscr{M}_{i_{a}}^{j_{a}}$-measurable with $i_{a}=a(\mu+\nu)+1$ and $j_{a}=a(\mu+\nu)+\mu$. Since *φ*-mixing random variables are strong mixing random variables and $\alpha(n)\leq\varphi(n)$, letting $V_{j}=\exp(itn^{-1/2}\eta_{j})$, by Lemma 5.4 we have

$$\begin{aligned}& \Biggl\vert E \bigl(\exp\bigl(itn^{-1/2}S_{n}^{\prime} \bigr) \bigr)-\prod_{j=0}^{k-1}E \bigl(\exp \bigl(itn^{-1/2}\eta_{j}\bigr) \bigr) \Biggr\vert \\& \quad \leq ck_{n}\varphi(\nu_{n}+1)\leq c\frac{n}{\mu_{n}+\nu_{n}} \frac{1}{\nu_{n}^{2}}\leq \frac{c}{\sqrt{nh^{d}}}\rightarrow0 \end{aligned}$$

by (5.19), (5.20), and the conditions $h_{n}\rightarrow0$ and $nh^{d}\rightarrow\infty$ as $n\rightarrow\infty$.

Third, we show (5.27), where $\eta_{j}$ is defined in (5.21). By the stationarity and (5.30) with $\mu_{n}$ replacing $\nu_{n}$, we have

5.34 $$ E\bigl(\eta_{j}^{2}\bigr)=\operatorname{Var}( \eta_{j})=\operatorname{Var}(\eta_{0})=\mu_{n} \sigma ^{2}(x) \bigl(1+o(1)\bigr),\quad 0\leq j\leq k-1, $$

so that

$$\frac{1}{n}\sum_{j=0}^{k_{n}-1}E\bigl( \eta_{j}^{2}\bigr)=\frac{k_{n}\mu_{n}}{n}\sigma ^{2}(x) \bigl(1+o(1)\bigr)\rightarrow\sigma^{2}(x), $$

since $k_{n}\mu_{n}/n\rightarrow1$.

Fourth, it is time to establish (5.28). Obviously, by (5.7) we obtain that

$$EZ_{i}^{2}=EZ_{1}^{2}\leq c_{1} \quad \text{and}\quad E|Z_{i}|^{2+\delta}= E|Z_{1}|^{2+\delta}\leq c_{2}\bigl(h^{d} \bigr)^{-\frac{\delta}{2}},\quad 1\leq i\leq n. $$

We can see that $\frac{\frac{1}{h^{d}}}{\mu_{n}}\leq\frac{c}{h^{d} \sqrt{\frac{n}{h^{d}}}}=\frac{c}{(nh^{d})^{\frac{1}{2}}}\rightarrow0$, since $nh^{d}\rightarrow\infty$ as $n\rightarrow\infty$. Therefore, by Lemma 5.3 with $\sum_{n=1}^{\infty}\varphi^{1/2}(n)<\infty$ we have that

5.35 $$\begin{aligned} E \Biggl\vert \sum_{i=1}^{\mu_{n}}(Z_{i}-EZ_{i}) \Biggr\vert ^{2+\delta} \leq&c_{1} \Biggl(\sum _{i=1}^{\mu_{n}}E|Z_{i}|^{2+\delta}+ \Biggl( \sum_{i=1}^{\mu _{n}}EZ_{i}^{2} \Biggr)^{\frac{2+\delta}{2}} \Biggr) \\ \leq& c_{2} \biggl(\mu_{n}\frac{1}{(h^{d})^{\frac{\delta}{2}}}+ \mu_{n}^{1+\frac{\delta}{2}}\biggr) \leq c_{3}\mu_{n}^{1+\frac{\delta}{2}}. \end{aligned}$$

Then, for all $\varepsilon>0$, by (5.34) and (5.35) it is easy to see that

$$\begin{aligned} E \bigl(\eta_{0}^{2}I\bigl(|\eta_{0}|\geq \varepsilon\sigma(x)n^{1/2}\bigr) \bigr) \leq&\frac{1}{(\varepsilon\sigma(x)n^{1/2})^{\delta}}E|\eta _{0}|^{2+\delta}I\bigl(|\eta_{0}|\geq \varepsilon \sigma(x)n^{1/2}\bigr) \\ \leq&\frac{1}{(\varepsilon\sigma(x)n^{1/2})^{\delta}}E|\eta _{0}|^{2+\delta}\leq c_{1}\frac{\mu_{n}^{1+\frac{\delta}{2}}}{n^{\frac{\delta}{2}}}. \end{aligned}$$

Similarly, for $0\leq j\leq k-1$, we get that

$$ E \bigl(\eta_{j}^{2}I\bigl(|\eta_{j}|\geq \varepsilon\sigma(x)n^{1/2}\bigr) \bigr)\leq c_{2} \frac{\mu_{n}^{1+\frac{\delta}{2}}}{n^{\frac{\delta}{2}}}. $$

Therefore, since $0<\delta\leq1$ and $nh^{d}\rightarrow\infty$, we obtain that, for all $\varepsilon>0$,

$$\begin{aligned} \frac{1}{n}\sum_{j=0}^{k-1}E \bigl( \eta_{j}^{2}I\bigl(|\eta_{j}|\geq\varepsilon \sigma(x)n^{1/2}\bigr) \bigr) \leq& \frac{ck\mu_{n}^{1+\frac{\delta}{2}}}{n^{1+\frac{\delta}{2}}} \leq \frac{c\mu_{n}^{1+\frac{\delta}{2}}\frac{n}{\mu_{n}+\nu_{n}}}{n^{1+\frac {\delta}{2}}} \leq\frac{c\mu_{n}^{\frac{\delta}{2}}}{ n^{\frac{\delta}{2}}} \\ =&c \biggl(\frac{\sqrt{\frac{n}{h^{d}}}}{n} \biggr)^{\frac{\delta}{2}}= \frac{c}{(nh^{d})^{\frac{\delta}{4}}} \rightarrow0. \end{aligned}$$

Therefore, (5.26), (5.27), and (5.28) hold for $S_{n}^{\prime}$, so that

5.36 $$ \frac{1}{\sqrt{n}}S_{n}^{\prime}\xrightarrow{~~\mathscr{D}~~}N \bigl(0,\sigma ^{2}(x)\bigr). $$

Consequently, (5.12) follows from (5.33) and (5.36). Finally, by (5.1), (5.2), and (5.12) we obtain (3.1). The proof of theorem is completed. □
